# Mannanase hydrolysis of spruce galactoglucomannan focusing on the influence of acetylation on enzymatic mannan degradation

**DOI:** 10.1186/s13068-018-1115-y

**Published:** 2018-04-19

**Authors:** Jenny Arnling Bååth, Antonio Martínez-Abad, Jennie Berglund, Johan Larsbrink, Francisco Vilaplana, Lisbeth Olsson

**Affiliations:** 1 Division of Industrial Biotechnology, Department of Biology and Biological Engineering, Chalmers University of Technology, 412 96 Gothenburg, Sweden; 2 Wallenberg Wood Science Center, Chalmers University of Technology, 412 96 Gothenburg, Sweden; 3 Division of Glycoscience, Department of Chemistry, School of Engineering Sciences in Chemistry, Biotechnology and Health, KTH Royal Institute of Technology, 100 44 Stockholm, Sweden; 4 Wallenberg Wood Science Center, School of Engineering Sciences in Chemistry, Biotechnology and Health, KTH Royal Institute of Technology, 100 44 Stockholm, Sweden; 5 Present Address: Department of Analytical Chemistry, Nutrition and Food Sciences, University of Alicante, 03690 Alicante, Spain

**Keywords:** Lignocellulose, Spruce, Galactoglucomannan, *Endo*-β-mannanases, GH5, GH26, *Cellvibrio japonicus*, Polysaccharide acetylation, Enzymatic degradation pattern, Acetyl esterases

## Abstract

**Background:**

Galactoglucomannan (GGM) is the most abundant hemicellulose in softwood, and consists of a backbone of mannose and glucose units, decorated with galactose and acetyl moieties. GGM can be hydrolyzed into fermentable sugars, or used as a polymer in films, gels, and food additives. *Endo*-β-mannanases, which can be found in the glycoside hydrolase families 5 and 26, specifically cleave the mannan backbone of GGM into shorter oligosaccharides. Information on the activity and specificity of different mannanases on complex and acetylated substrates is still lacking. The aim of this work was to evaluate and compare the modes of action of two mannanases from *Cellvibrio japonicus* (*Cj*Man5A and *Cj*Man26A) on a variety of mannan substrates, naturally and chemically acetylated to varying degrees, including naturally acetylated spruce GGM. Both enzymes were evaluated in terms of cleavage patterns and their ability to accommodate acetyl substitutions.

**Results:**

*Cj*Man5A and *Cj*Man26A demonstrated different substrate preferences on mannan substrates with distinct backbone and decoration structures. *Cj*Man5A action resulted in higher amounts of mannotriose and mannotetraose than that of *Cj*Man26A, which mainly generated mannose and mannobiose as end products. Mass spectrometric analysis of products from the enzymatic hydrolysis of spruce GGM revealed that an acetylated hexotriose was the shortest acetylated oligosaccharide produced by *Cj*Man5A, whereas *Cj*Man26A generated acetylated hexobiose as well as diacetylated oligosaccharides. A low degree of native acetylation did not significantly inhibit the enzymatic action. However, a high degree of chemical acetylation resulted in decreased hydrolyzability of mannan substrates, where reduced substrate solubility seemed to reduce enzyme activity.

**Conclusions:**

Our findings demonstrate that the two mannanases from *C. japonicus* have different cleavage patterns on linear and decorated mannan polysaccharides, including the abundant and industrially important resource spruce GGM. *Cj*Man26A released higher amounts of fermentable sugars suitable for biofuel production, while *Cj*Man5A, producing higher amounts of oligosaccharides, could be a good candidate for the production of oligomeric platform chemicals and food additives. Furthermore, chemical acetylation of mannan polymers was found to be a potential strategy for limiting the biodegradation of mannan-containing materials.

**Electronic supplementary material:**

The online version of this article (10.1186/s13068-018-1115-y) contains supplementary material, which is available to authorized users.

## Background

Climate change, increasing energy needs, and decreasing oil resources call for a shift away from our dependency on fossil fuels toward renewable fuels from biomass [1]. Softwood biomass is available in large quantities, and has great potential as a raw material for the production of not only biofuels, but also chemicals and biomaterials; moreover, its utilization does not compete with food production [2, 3]. Norway Spruce (*Picea abies*) is the major source of softwood in Northern Europe and is a promising feedstock for biorefineries [4]. Cellulose is the main component of softwood biomass and has traditionally been used in pulping. However, in order to exploit biomass more fully in a biorefinery, utilization of the hemicelluloses is required [5]. Hemicelluloses can be hydrolyzed into fermentable sugars, but may also serve as oligomeric and polymeric starting materials for the manufacture of high-value products such as films, coatings, gels, food additives, prebiotics, and biodegradable components in composite materials [4, 6–9]. Enzymes with high specificities can be important in degrading and modifying hemicelluloses for different purposes.

**Fig. 1:**
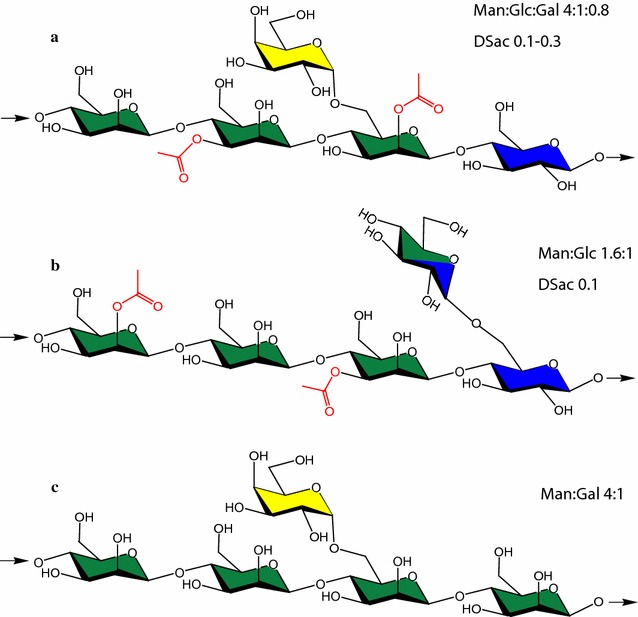
Chemical structures: **a** spruce *O*-acetyl-galactoglucomannan (SpGGM), **b** konjac glucomannan (KGM), and **c** locust bean gum galactomannan (LBG). C2 and C3 are potential acetylation sites (marked in red) on the SpGGM and KGM mannose units. Average monosaccharide ratios of mannose (green), glucose (blue), and galactose (yellow), and degree of substitution by acetylation (DSac) are shown for each mannan. The degree of substitution on glucose or mannose units in KGM is low (8%). KGM is therefore referred to as a linear polysaccharide, compared with the highly substituted LBG and SpGGM

The primary hemicellulose in softwoods is *O*-acetyl-galactoglucomannan (GGM), representing approximately 20% of the dry weight. GGM consists of a backbone of β-(1 → 4)-d-mannopyranosyl and β-(1 → 4)-d-glucopyranosyl units decorated with single α-(1 → 6)-linked d-galactopyranosyl units attached solely to the mannopyranosyl units. The typical Man:Glc:Gal ratio in Norway spruce GGM (SpGGM) has been reported to be 3.5–4.5:1:0.5–1.1, with the mannose C2 and C3 units *O*-acetylated typically at a degree of 0.2–0.3 [10–13] (Fig. 1). Variations in these structures depend on both the extraction method and the raw material itself [5, 13–15]. Mannan polysaccharides are found in the cell walls of most plants, and may also serve as storage polysaccharides in certain species, e.g., the tubers of konjac or locust seeds (Fig. 1). Konjac glucomannan (KGM) and locust bean gum galactomannan (LBG) have been commercialized and utilized for several of the potential applications mentioned above [5, 7]. KGM consists of a backbone of β-(1 → 4)-linked d-mannopyranosyl and d-glucopyranosyl units at a Glc:Man ratio of 1:1.6 [16], and has a low degree (8%) of branching to the backbone glucosyl units, through β-(1 → 6)-linked glucosyl or mannosyl units [17]. The backbone of LBG consists of β-(1 → 4)-linked d-mannopyranosyl units, branched with α-(1 → 6)-linked d-galactopyranosyl units [18], with a Gal:Man ratio of around 1:4. LBG is not acetylated, while KGM is naturally acetylated to a low degree (~ 0.1). They can both serve as model substrates for chemical acetylation [19, 20].

The highly decorated and acetylated SpGGM requires a range of enzymes for complete hydrolysis into monosaccharides, including *endo*-β-mannanases (EC 3.2.1.78), β-mannosidases (EC 3.2.1.25), β-glucosidases (EC 3.2.1.21), α-galactosidases (EC 3.2.1.22), and acetyl esterases (EC 3.1.1.72). *Endo*-acting β-mannanases, henceforth referred to as mannanases, play a vital role in mannan degradation, by hydrolyzing the β-d-1,4 linkages between mannose residues in the polysaccharide backbone, producing smaller mannooligosaccharides [21]. Mannanases release mainly mannobiose and mannotriose as end products, and exhibit open cleft-shaped active sites able to accommodate up to 5–6 hexose units [22, 23]. Based on the amino acid sequences, mannanases have been described in three glycoside hydrolase (GH) families in the Carbohydrate-Active enZYmes database (CAZy) [24, 25]: GH5, GH26 and recently also GH113 [26]. The majority of the characterized mannanases have been classified into the GH5 and GH26 families [26, 27], which both belong to the glycoside hydrolase clan GH-A. GH-A enzymes share the TIM (triose phosphate isomerase) (β/α)_8_ barrel fold and a retaining reaction mechanism [28].

A number of studies have described GH5 and GH26 mannanases, reporting differences in substrate specificities, substrate degradation patterns, and modes of action, in addition to different proposed biological roles of the enzymes [21, 22, 29–31]. For instance, *Cj*Man5A from the bacterium *Cellvibrio japonicus* has been suggested to target plant cell walls, due to the presence of several carbohydrate binding modules, while *Cj*Man26A from the same organism has been shown to be more active on shorter mannooligosaccharides [29, 30]. In contrast, a comparison of GH5 and GH26 mannanases from the fungus *Podospora anserina* showed the opposite behavior, namely that *Pa*Man26A generated longer oligosaccharides that could be further processed by *Pa*Man5A [22]. Regarding substrate specificity, it has been reported that bacterial GH5 mannanases have a more promiscuous specificity for glucose and mannose units at the − 2 and + 1 subsites, while the investigated GH26 mannanases exhibit a higher specificity for mannose at the − 2 subsite [21, 29]. Restriction of mannanase action due to galactose substituents has previously been observed in certain enzymes [32–34], though mannanases insensitive to galactosylation, able to efficiently degrade highly substituted galactomannans, have also been described [31, 34]. Von Freiesleben et al. [31] have characterized two fungal GH26 mannanases that form α-galactosyl-mannose as the main degradation product, suggesting a capability to accommodate galactopyranosyl residues at both subsites + 1 and − 1.

Few comparative studies on mannanase activity and specificity on different mannan substrates have been published. Studies regarding functional differences in mannanases able to hydrolyze softwood biomass such as SpGGM are especially lacking, and the degradation patterns to date are mainly hypothetical [35, 36]. In order to better understand how the action of mannanases differs, further studies of mannanases from different families of different origins on a variety of well-defined mannan substrates are required. In this study, we set out to expand the current knowledge on substrate specificities, by investigating how mannan substrates with distinct backbone structures, decorated to different degrees (by both galactosylation and acetylation), affect the action of a GH5 and a GH26 mannanase from *C. japonicus* (*Cj*Man5A and *Cj*Man26A). In addition to naturally acetylated SpGGM, chemically acetylated and native KGM and LBG were used as substrates. Enzyme action and cleavage patterns were assayed with advanced analytic techniques, including anion-exchange chromatography and mass spectrometry, to study the effect of substrate structure on the hydrolyzability of the substrates and the product profiles of these enzymes.

## Methods

Ultrapure water, purified in a Milli-Q system (Millipore) to a resistivity of *ρ* > 18.2 MΩ cm, was used in all experiments.

### Substrates

Native locust bean gum galactomannan (LBG_N_) (from *Ceratonia siliqua* seeds, Sigma Aldrich, Stockholm, Sweden) and native konjac glucomannan (KGM_N_) (Konson Konjac Gum Co., Ltd, Wuhan, China) were chemically acetylated to different degrees (as described below). Table 1 depicts the distributions of monosaccharides and the DSac of the two purchased substrates. The sugar composition was measured by high-performance anion-exchange chromatography equipped with pulsed amperometric detection (HPAEC-PAD) (as described below). All substrates were dissolved in water to a concentration of 0.2% (w/v).

**Table 1:** Compositions and molecular structures of the substrates used in this study

Sample	DSac^a^	MW (kDa)	Carbohydrate composition %^b^							
			Ara	Gal	Glc	Xyl	Man	Rha	GalA	GlcA
KGM_N_	0.09	1000	0.1	0.5	38.9	0.2	60.3	–	–	–
KGM_A_	0.7									
KGM_B_	2.1									
LBG_N_	0	1000	1.6	20.7	2.7	0.5	74.5	–	–	–
LBG_A_	0.8									
LBG_B_	1.9									
SpGGM	0.13	30	1.3	11.3	20.8	8.6	53.1	1.5	3.2	0.3

^a^ Degree of acetylation calculated by saponification and quantification of acetic acid using HPLC. Four individual measurements were made, with a standard deviation of < 10%

^b^ Carbohydrate composition (mol%), quantified with HPAEC-PAD. Duplicate measurements were made, with a standard deviation of < 7%, except for KGM Xyl, which had a standard deviation of 48%

SpGGM was extracted using pressurized hot-water extraction at 170 °C under similar conditions to those reported by Song et al. [37]. In brief, the fiberized spruce wood was extracted in an accelerated solvent extraction system (ASE-300, Dionex, Sunnyvale, CA, USA) with buffered water at pH 5 (0.2 M formate buffer), at 170 °C for 20 min. After extraction, the samples were dialyzed, freeze-dried, and purified enzymatically with β-xylanase treatment. The polysaccharide composition (mass %) of the final SpGGM fraction was 86.5% galactoglucomannan, 8.68% arabinoglucuronoxylan, and 4.83% pectin. The monosaccharide composition and DSac values are presented in Table 1.

### Analysis of substrate composition

The sugar compositions of LBG_N_, KGM_N_, and SpGGM were determined after acid hydrolysis using the SCAN-CM 71:09 method, and analyzed in duplicate using an ICS-3000 HPAEC-PAD system (Dionex, Sunnyvale, CA, USA) equipped with a Dionex CarboPac PA1 column, as described by McKee et al. [38]. Saponification and analysis of the acetic acid content by high-performance liquid chromatography (HPLC) were used to determine DSac, as described by Bi et al. [20]. The system used was an Ultimate-3000 HPLC system (Dionex, Sunnyvale, CA, USA) equipped with a Phenomenex Rezex ROA-Organic acid column.

### Acetylation

Chemical acetylation of KGM_N_ and LBG_N_ was performed using two different methods to obtain various degrees of substitution. To obtain a low DSac, a method described previously [19] was used. In short, 1 g of polysaccharide was dissolved in 50 mL acetic anhydride and 1 mL 50% (w/w) NaOH, and incubated for 5–7 h at 120 °C. The KGM_N_ sample was pretreated in 10 mL 50% (v/v) acetic acid and dried prior to acetylation. The fractions with low acetyl substitution obtained from this treatment are denoted KGM_A_ and LBG_A_, and DSac was determined to be 0.7 and 0.8 for the respective samples (Table 1).

The fractions with higher DSac were dissolved in formamide and pyridine, and acetylated with acetic anhydride, as described by Bi et al. [20]. When a total amount of 6.6 mL acetic anhydride had been added, the DSac was 2.1 for KGM and 1.9 for LBG. These samples are denoted KGM_B_ and LBG_B_. Naturally acetylated SpGGM has acetyl groups at the C2 and C3 positions [5] but chemically, the C6 position can also be acetylated [13]. Since polymeric hexoses have three possible acetylation sites, a value of DSac of 3 indicates complete acetylation.

### Substrate solubility measurements

A gravimetric method was used determine how the solubility of the mannan substrates was affected by chemical acetylation. Two millilitre of each mannan substrate: LBG_N_, KGM_N_, LBG_A_, KGM_A_, LBG_B_ and KGM_B_, was dissolved in water to a concentration of 0.2% (w/v), and then centrifuged for 15 min at 14,500 rpm. The resulting supernatant was regarded as the soluble fraction, and the remaining pellet as the insoluble fraction. Both fractions were dried in a vacuum oven at 40 °C overnight, and then weighed. The mass % of the soluble and insoluble fractions was calculated based on the total mass of the two fractions. DSac was determined for the soluble and insoluble fractions of both LBG_A_ and KGM_A_, as described above.

### Enzymes

Two mannanases from *C. japonicus* were used in this study: *Cj*Man5A (CZ0055, Nzytech, Lisbon, Portugal) and *Cj*Man26A (E-BMACJ, Megazyme, County Wicklow, Ireland). For synergy studies, a carbohydrate esterase family 2 acetyl xylan esterase from *Clostridium thermocellum* (*Ct*Axe2A) (CZ00321, Nzytech, Lisbon, Portugal), with reported activity on acetylated glucomannan and galactomannan was used. All enzymes were diluted in 100 mM sodium phosphate buffer, pH7.

### Enzymatic hydrolysis

Enzymatic hydrolysis of mannan substrates was performed at 35 °C in a thermomixer, with enzyme concentrations of 10, 25 and 100 nM. The reactions were incubated with 0.1% (w/v) KGM_N_, KGM_A_, LBG_N_, LBG_A_, or SpGGM, in a 25 mM sodium phosphate buffer, pH 7 for 24 h. Samples were collected at several times for the analysis of the reducing sugars released, and on three occasions for qualitative and quantitative product analysis with HPAEC-PAD. Samples were also collected after 24 h for further analysis with electrospray ionization mass spectrometry (ESI–MS) and size exclusion chromatography (SEC). The sampling times and enzyme concentrations were chosen based on the reducing sugar reaction profiles, so as to obtain samples with a low degree of hydrolysis, a moderate degree of hydrolysis and complete hydrolysis (with 100 nM enzyme concentration). The reactions were stopped by heating at 95 °C for 20 min.

### Enzymatic hydrolysis with the addition of acetyl xylan esterase

Hydrolysis reactions containing 0.1% (w/v) KGM_N_ or SpGGM in 25 mM sodium phosphate buffer, pH7, were incubated with 10 nM *Cj*Man5A or *Cj*Man26A, together with high (50 nM), low (5 nM) or no *Ct*Axe2A, respectively, for 6 h. Synergetic effects between *Ct*Axe2A and the mannanases were analyzed by quantification of the reducing sugars released.

### Detection of reducing sugars

Prior to the detection of reducing sugars, the enzymatic reactions were stopped by the addition of an equal volume of DNS (3,5-dinitrosalicylic acid) reagent [39]. The mixture was heated for 15 min at 85 °C, and 100 μL was transferred to a microplate. The amounts of reducing sugar equivalents were measured at 595 nm, in a spectrophotometer (FLUOstar Omega, BMG LABTECH, Offenburg, Germany). Mannose was used to generate standard curves.

### Size exclusion chromatography

The molecular weight distribution of KGM_N_ and LBG_N_, before and after enzymatic hydrolysis, was obtained by measuring the apparent molecular weight of the polymers with SEC. The substrates were hydrolyzed in water instead of buffer solution to avoid interference in the SEC analysis. Samples were filtered with 0.2 µm nylon syringe filters before injection into the Dionex Ultimate-3000 HPLC system equipped with a guard column and three PSS Suprema columns connected in series (pore sizes 30, 1000 and 1000 Å, particle size 10 µm). A Waters-2414 refractive index detector was used (Waters, Milford, MA, USA). A calibration curve was performed with Pullulan Standards with molar masses ranging between 342 and 708,000 Da (PSS, Mainz, Germany). A NaOH solution (10 mM) was used as mobile phase, at a flow rate of 1 mL/min, and the oven was set at 40 °C.

### Identification and quantification of oligosaccharides by HPAEC-PAD

Mannooligosaccharides were analyzed with HPAEC-PAD on an ICS-3000 system, equipped with a 4 × 250 mm Dionex Carbopac™ PA200 column and a 4 × 50 mm guard column (Dionex, Sunnyvale, CA, USA). Analysis was performed at 30 °C at a flow rate of 0.5 mL/min with injection volumes of 10 μL. The eluents were A: water, B: 300 mM sodium hydroxide, and C: 1 M sodium acetate. Elution was carried out isocratically with 85% A and 15% B for the first 15 min, followed by a 15-min gradient to 33% B and a 20-min gradient to 33% B + 10% C. At 40 min, a 10-min ramp to 66% C was performed as a cleaning step. After elution, the column was regenerated for 10 min using the starting conditions, 85% A and 15% B [40]. Mannose (M1), mannobiose (M2), mannotriose (M3), mannotetraose (M4), and mannopentaose (0.002–0.1 g/L) were used as standards. All standards and samples were spiked with 0.1 g/L fucose as an internal standard. The peak areas of the analytes were divided by the corresponding peak area of the internal standard before being quantified against standard curves.

### Analysis of the acetylation pattern of the reaction products

The acetylation patterns of mannooligosaccharide products were analyzed with ESI–MS. The hydrolysates were desalted with HyperSep™ Hypercarb™ solid phase extraction cartridges (Thermo Fischer, UK). Positive-ion ESI–MS was performed on a Q-TOF^2^ ESI mass spectrometer (Micromass, Wilmslow, UK). Samples were dissolved in acetonitrile (50%) containing 0.1% formic acid, and infused directly into the mass spectrometer through the capillary liquid chromatography module at a rate of 8 μL/min. The ESI source was operated at 3.3 kV with a desolvation temperature of 140 °C and a cone voltage of 70–80 V. The oligosaccharides were detected as [M+Na]^+^ adducts.

## Results

The primary aim of this study was to investigate the action of mannanases on the heterogeneous, branched, and naturally acetylated polymer SpGGM. Two mannanases from *C. japonicus*, *Cj*Man5A and *Cj*Man26A, were selected and used to hydrolyze three native mannan substrates: KGM_N_, LBG_N_, and SpGGM. KGM_N_ and LBG_N_ serve as model substrates as they share important structural and chemical features with SpGGM, but also are well investigated and understood. The enzymatic activity and cleavage patterns of the substrates were evaluated by detection of the reducing sugars, SEC, HPAEC-PAD and ESI–MS. The chemically acetylated substrates with medium (KGM_A_, LBG_A_) and high DSac (KGM_B_, LBG_B_) were also included in the study to investigate the role of acetylation on the mannanase action. We were unable to determine the exact positions of the acetyl groups on the chemically modified substrates using NMR spectroscopy, due to the poor solubility of them in DMSO and chloroform. The chemical acetylation method employed in this study is not regioselective, i.e., acetylation at either C2, C3 or C6 is possible. We anticipate that acetylation of the primary hydroxyl group of C6 is favoured, while the C2 and C3 hydroxyls may have similar reactivity. This has previously been observed, where applying the same chemical procedure on a soluble oligosaccharide substrate, cellobiose, indeed resulted in acetylation of all available positions: C2, C3, and C6 [20].

### Actions of *Cj*Man5A and *Cj*Man26A on different mannan substrates

**Fig. 2:**
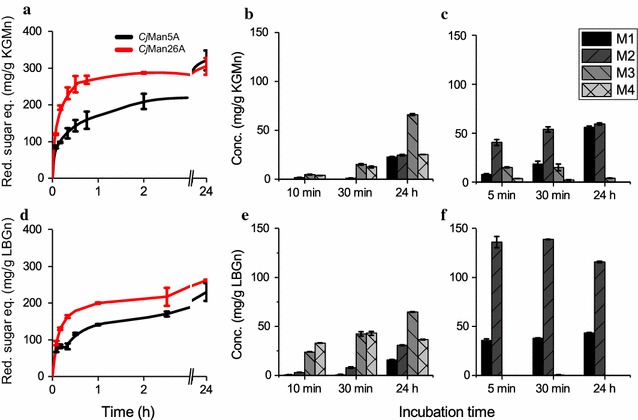
Temporal pattern showing the increase in reducing sugar equivalents over 24 h when applying *Cj*Man5A or *Cj*Man26A on KGM_N_ (**a**) and LBG_N_ (**d**). The amounts of mannooligosaccharides produced (M1–M4) at three times during hydrolysis with either *Cj*Man5A (**b** and **e**) or *Cj*Man26A (**c** and **f**) on KGM_N_ (upper panel) and LBG_N_ (lower panel) are also shown. The hydrolysis reactions analyzed in terms of reducing sugars contained 10 nM enzyme and 0.1% (w/v) mannan substrate. The reaction times and enzyme concentrations used for the quantification of mannooligosaccharides using HPAEC-PAD were for *Cj*Man5A reactions: 10 min, 25 nM, 30 min, 25 nM, and 24 h, 100 nM; and for *Cj*Man26A reactions: 5 min, 10 nM, 30 min, 25 nM, and 24 h, 100 nM. The high enzyme concentration of 100 nM was used for 24-h reactions to ensure complete hydrolysis. Error bars signify standard errors of the mean of duplicate HPAEC-PAD measurements or triplicate reducing sugar measurements

In order to investigate the time course of mannan hydrolysis for each mannanase, the two enzymes were incubated individually with the native substrates, and the reaction products were monitored by quantification with the DNS reagent of the reducing ends generated. The profiles showed that hydrolysis using *Cj*Man26A reached a product plateau earlier than when using *Cj*Man5A on the more linear substrate KGM_N_ (Fig. 2a). However, both enzymes exhibited similar rate profiles when acting on the galactose-containing substrates LBG_N_ (Fig. 2d) and SpGGM (see Additional file 1: Figure S1). Both mannanases gave similar conversion yields after 24-h hydrolysis of KGM_N_. For the substrates containing galactose side chains, LBG_N_ and SpGGM, hydrolysis by *Cj*Man26A resulted in a slightly higher final conversion. It is important to note that these enzymes produce a range of oligosaccharides of various lengths and compositions on the different mannan polysaccharides, leading to various amounts of reducing sugars. The results regarding the release of reducing sugars therefore only give an overall picture of the differences in enzymatic substrate hydrolyzability.

To determine whether the enzymes were able to completely convert the mannan polysaccharides into oligosaccharides, and to determine the size distribution of the products (MW > 340 Da), SEC was performed on the reaction products after the incubation of KGM_N_ and LBG_N_ for 24 h (see Additional file 2: Figure S2). The SEC profiles for KGM_N_ and LBG_N_ before mannanase treatment showed a substrate peak at 17 min (average MW ~ 860 kDa; Pullulan Standards), which shifted toward lower molecular weight after incubation with *Cj*Man5A and *Cj*Man26A, demonstrating degradation of the mannan polymers into oligosaccharides. *Cj*Man5A produced oligosaccharides distributed around 900 Da from KGM_N_ and around 1500 Da from LBG_N_, corresponding to approximately five and eight hexoses, respectively. *Cj*Man26A, on the other hand, produced oligosaccharides with a MW of around 1000 Da (six hexoses) from KGM_N_, while hydrolysis of LBG_N_ showed one peak at 1800 Da (ten hexoses) and one at 500 Da (three hexoses).

**Fig. 3:**
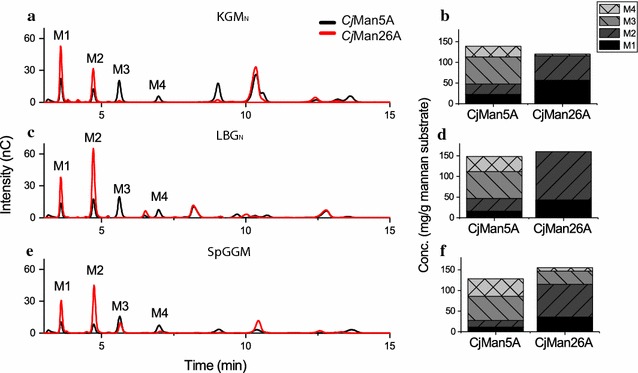
Oligosaccharide product profiles obtained from HPAEC-PAD analysis of hydrolysis reactions (left panel), and quantification of M1–M4 oligosaccharides (right panel) after 24-h reactions with 100 nM *Cj*Man5A or *Cj*Man26A on KGM_N_ (**a**, **b**), LBG_N_ (**c**, **d**) and SpGGM (**e**, **f**). The profiles show that the two enzymes produced different amounts of the linear mannooligosaccharides M1–M4, as well as different types of complex oligosaccharides on the three substrates. M1 and M2 were the major mannans produced by *Cj*Man26A, with a proportionally higher amount of M2 for the branched LBG_N_ and SpGGM, while M1 was more dominant in the hydrolysis of KGM_N_. *Cj*Man26A also generated a considerable amount of M3 on SpGGM. Hydrolysis with *Cj*Man5 produced only small amounts of M1 and M2, while M3 and M4 were the main mannooligosaccharides produced. Duplicate measurements were performed, with standard errors of the mean of < 3%

To obtain detailed information on the differences in the smaller oligosaccharides produced by each enzyme, in terms of the distribution and quantities, HPAEC-PAD was performed on the reaction mixtures at three different incubation times (5/10 min, 30 min, 24 h). The product profiles showed variations in both the distribution and quantities of the linear mannooligosaccharides M1–M4 over time, depending on which mannanase had been used for hydrolysis (Fig. 3a–d). (The HPAEC-PAD spectra for all three incubation times are given in Additional file 3: Figure S3).

Hydrolysis of KGM_N_ by *Cj*Man5A led to increasing concentrations of M1–M4 over time, although M1 and M2 could not be detected until after 30 min, and M3 showed the highest concentration after 24 h. During hydrolysis with *Cj*Man26A, M1–M3 were detected much earlier after 5 min, and increased levels of M1 and M2 were observed over time. M3 and M4 concentrations peaked after 30 min, but were not detectable after 24 h, likely due to their further undergoing hydrolysis into M1 and M2.

Using the decorated LBG_N_ substrate, predominantly M3 and M4 were produced by *Cj*Man5A initially, but between 30 min and 24 h, increasing levels of M1–M3 were observed. The product pattern for the hydrolysis of LBG_N_ by *Cj*Man26A was completely different; high amounts of M1 and M2 being produced already after 5 min, and M1 continually increasing over time. Only trace amounts of M3 and M4 were detected, if detected at all. In conclusion, the concentration of shorter mannooligosaccharides increased with time regardless of which enzyme was employed, but *Cj*Man26A produced significantly higher amounts of M1 and M2. Furthermore, *Cj*Man26A was more efficient in the initial stages of hydrolyzation, in agreement with the reducing sugar measurements, producing high amounts of short oligosaccharides, especially on the LBG_N_ substrate.

Due to the limited availability of mannooligosaccharide standards and co-elution of heterogeneous oligosaccharide products, the HPAEC-PAD analysis provided no quantitative information on longer mannooligosaccharides or more heterogeneous products including glucose and galactose units. However, the measurements gave interesting information on the differences between the shortest end products produced by *Cj*Man5A and *Cj*Man26A hydrolysis and on the differences in preference regarding oligosaccharide backbone compositions and side groups of the two enzymes.

End-point hydrolysis reactions (24 h) were also performed on the native SpGGM substrate, which shares the mannose–glucose-containing backbone with KGM_N_, but is decorated with galactose units, like LBG_N_. HPAEC-PAD profiles of 24-h reactions on KGM_N_, LBG_N_ and SpGGM are shown in Fig. 3a, c, e together with the quantities of M1–M4 (Fig. 3b, d, f) to visualize the variety of shorter mannooligosaccharide products generated with the various substrates and enzymes. These profiles clearly show different cleavage patterns. Interestingly, the hydrolysis of SpGGM showed some common features with the hydrolysis of KGM_N_ and LBG_N_. In general, SpGGM was mainly hydrolyzed into M3 and M4 by *Cj*Man5A, while *Cj*Man26A was capable of producing a larger quantity of the smaller products M1 and M2, as observed for the substrates KGM_N_ and LBG_N_. The ratio between the amounts of M1 and M2 produced by *Cj*Man26A on SpGGM was more similar to that on LBG_N_ than on KGM_N,_ i.e., a higher amount of M2 than M1. However, *Cj*Man26A also produced high concentrations of M3 and M4, oligosaccharides that were not observed to the same extent in the hydrolysis of either KGM_N_ or LBG_N_. The hydrolysis of SpGGM showed an unidentified product peak eluting at 9 min, and another after 10 min, similar to the KGM_N_ hydrolysis profile. These peaks probably correspond to larger oligosaccharides that contain glucose. These peaks were not observed in the LBG_N_ profile, where instead four smaller peaks eluted between 9 and 11 min, probably corresponding to galactose-containing mannooligosaccharides.

### The effects of chemical and native acetylation on substrate hydrolyzability

To determine whether varying degrees of acetylation of mannans significantly affected their enzymatic hydrolyzability by mannanases, hydrolysis reactions were performed on chemically acetylated (KGM_A_ and LBG_A_, DSac 0.7–0.8) and native (KGM_N_ and LBG_N_) substrates, and the amounts of reducing sugars released were analyzed (see Additional file 4: Figure S4). Hydrolysis of KGM_A_ generated about 20% lower amounts of reducing sugar equivalents (mg/g substrate) than the hydrolysis of native KGM_N_. For the decorated LBG_A_, no reducing sugars were detected in standard reactions (10 nM enzyme), and even with tenfold increased enzyme concentrations, lower hydrolyzability was observed than with the standard reactions on the native and non-acetylated LBG_N_. The highly acetylated mannans (KGM_B_ and LBG_B_) with a DSac > 1.9 could not be hydrolyzed into detectable concentrations of reducing sugars, even at enzyme concentrations of 100 nM and prolonged incubation time. Moreover, the product patterns and quantification of M1–M4 products obtained with HPAEC-PAD showed a clear correlation between a high degree of acetylation and low hydrolyzability (see Additional file 5: Figure S5 and Additional file 6: Figure S6). These observations indicate that high degrees of both acetylation and galactosyl substitution reduced the enzymatic hydrolysis of the mannans by both *Cj*Man5A and *Cj*Man26A.

To elucidate whether the low hydrolyzabilities of the KGM and LBG substrates were due to a reduction in solubility resulting from the chemical acetylation (as judged by visual inspection of the dissolved substrates), the soluble and insoluble fractions were analyzed gravimetrically for both the native and the acetylated substrates. The results showed a clear decrease in solubility upon acetylation (Table 2). When acetylated to a degree of 0.7–0.8, the soluble fraction was reduced by 38% in the case of LBG_A_ and by 43% for KGM_A_. The highly acetylated substrates KGM_B_ and LBG_B_ (DSac ~ 2) only contained about 15% soluble material. The degree of acetylation was measured for each fraction of the acetylated KGM_A_ and LBG_A_ substrates, showing that practically all the acetyl groups were present in the insoluble fraction, indicating an uneven distribution of acetylated moieties on the polysaccharides. The hydrophobization of the mannan substrates introduced by acetylation is probably the reason for the reduced solubility of the chemically acetylated mannans, which in turn resulted in reduced hydrolyzability and an apparent reduction in mannanase activity.

**Table 2:** Mass % and DSac values of the soluble and insoluble fractions of native and chemically acetylated substrates

Sample	Total fraction	Soluble fraction		Insoluble fraction	
	DSac^a^	Mass %^b^	DSac^c^	Mass %^d^	DSac^e^
LBG_N_	0	66 ± 8.7	NM	34 ± 8.7	NM
KGM_N_	0.09	84 ± 4.9	NM	16 ± 4.9	NM
LBG_A_	0.8	41 ± 3.1	0	59 ± 3.1	1.5
KGM_A_	0.7	48 ± 6.3	0.1	52 ± 6.3	1.8
LBG_B_	1.9	13 ± 2.7	NM	87 ± 2.7	NM
KGM_B_	2.1	15 ± 4.3	NM	85 ± 4.3	NM

*NM* not measured

^a^ Four individual measurements were made with standard deviations of < 10%

^b,d^ Errors represent standard deviations of triplicate measurements

^c,e^ Triplicate measurements were made with standard deviations of < 17%

To determine whether the insoluble fractions of KGM_A_ and LBG_A_ were more resistant to enzymatic hydrolysis than the soluble fractions, overnight hydrolysis of each fraction with *Cj*Man5A or *Cj*Man26A was performed, followed by quantification of the reducing sugars. From these experiments, it was evident that the insoluble and highly acetylated fractions were essentially unaffected by the mannanases, with insoluble KGM releasing a mere 10% of reducing sugar equivalents (mg/g substrate) compared with the soluble substrate, and no reducing sugars were released at all after enzyme treatment of the insoluble LBG_A_ fraction.

**Fig. 4:**
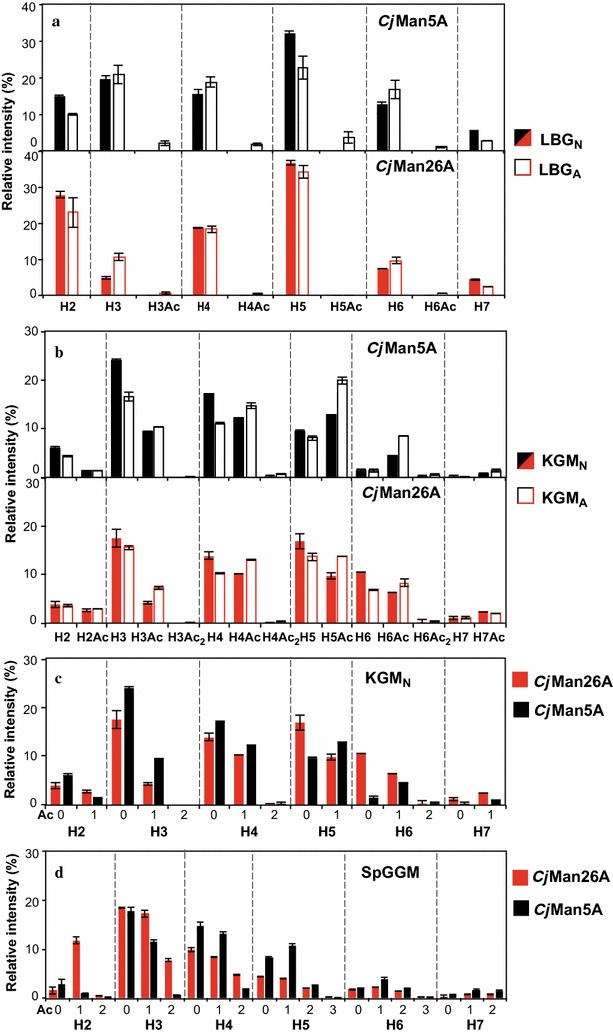
Oligomeric mass profiling (OLIMP) of the different substrates after 24-h hydrolysis with the two mannanases. **a** Comparison of native and chemically acetylated locust bean galactomannan (LBG_N_ and LBG_A_). **b** Comparison of native and chemically acetylated konjac glucomannan (KGM_N_, KGM_A_). **c** Comparison of mannanase action on native acetylated konjac glucomannan (KGM_N_). **d** Comparison of mannanase action on native acetylated spruce galactoglucomannan (SpGGM). Enzymatic hydrolysis was performed using 100 nM *Cj*Man5A or *Cj*Man26A. The error bars show the standard deviations of duplicate measurements. H refers to the number of hexoses in the mannooligosaccharides (Man, Glc or Gal), and Ac refers to the number of acetylations

The oligomeric mass profiles of the hydrolytic products (obtained with ESI–MS) after 24-h incubation of KGM and LBG (native and acetylated) with either *Cj*Man5A or *Cj*Man26A were compared. Oligomeric mass profiling (OLIMP) measures the relative peak heights within each spectrum of released oligosaccharides, and provides information about oligosaccharide length and the presence of acetyl groups (Fig. 4). The mass spectra indicate the number of acetyl groups (Ac) on the released hexooligosaccharides (H2–H7) after mannanase treatment, and show which acetylated products are generated from the acetylated substrates with which mannanase. However, it is unfortunately not possible to distinguish the nature of the oligosaccharides in terms of Man, Glc and Gal content from these spectra. Neither was it possible to assign the regiochemistry of the acetyl groups to each sugar unit from the mass spectra. This is indeed a very complex analytical question due to the instability of the acetyl groups and the isobaric nature of the sugar monomers (Glc, Man, and Gal) in the mannan oligosaccharides, as it has been recently addressed by Liu et al. [41] using LC–ESI–MS/MS. The studies of the intramolecular positioning of the acetylations within the mannan oligosaccharides require specific method development and are outside of the scope of this study.

Investigating the hydrolysis of LBG_N_ showed that *Cj*Man26A was capable of releasing shorter oligosaccharides than *Cj*Man5A, in agreement with the results of the HPAEC-PAD analyses (Fig. 4a). Chemical acetylation of LBG_A_ inhibited enzymatic action, as discussed above, and a tenfold increase in enzyme concentration was required to detect hydrolysis by both *Cj*Man26A and *Cj*Man5A. With this higher enzyme concentration, small amounts of acetylated oligosaccharides were observed in the OLIMP of LBG_A_, in particular with *Cj*Man5A. However, acetylation resulted in a small relative reduction in the amount of H2 oligosaccharides and a slight increase in the relative *intensities* for some of the peaks of the longer oligosaccharides (H3–H7) for LBG_A_ compared to LBG_N_.

For KGM_N_, the presence of Glc in the mannan backbone affected the OLIMP with both *Cj*Man26A and *Cj*Man5A (Fig. 4b), compared with LBG_N_, in agreement with the product quantification by HPAEC-PAD (Fig. 3). A substantially lower amount of H2 was produced by *Cj*Man26A after the hydrolysis of KGM_N_ than LBG_N_. Also, higher relative amounts of shorter oligosaccharides (H2 and H3) could be detected by ESI–MS after hydrolysis with *Cj*Man5A, compared to *Cj*Man26A, in agreement with the more promiscuous specificity for Glc and Man units in the backbone previously reported for *Cj*Man5A [29]. KGM_N_ is naturally acetylated, with a reported DSac of 0.09. Acetylated hexooligosaccharide products were therefore observed for the native KGM_N_, but the intensities of the peaks were slightly higher for the chemically acetylated KGM_A_. This indicates that both enzymes were able, at least partially, to hydrolyze the highly acetylated and insoluble fractions of the KGM_A_ and LBG_A_ substrates. Comparison of the OLIMP profiles after the hydrolysis of native KGM_N_ with the two enzymes (Fig. 4c) shows that higher relative intensities of the acetylated oligosaccharide peaks were obtained with *Cj*Man5A than with *Cj*Man26A, which may indicate a higher tolerance of the former to acetylation.

The SpGGM extracted for this work was acetylated to a DSac of 0.13 and contains a mixed Glc/Man backbone similar to KGM_N_, but is additionally substituted with Gal moieties. The relatively low number of acetylations on the SpGGM (compared to extracted SpGGM reported previously [11–15]) enables comparison of the two mannanase enzymes on a linear and a branched mannan polymer with similar DSac and backbone structure. The acetylation patterns of oligosaccharides (degree of polymerization from 2 to 7) resulting from the hydrolysis of SpGGM are shown in Fig. 4d. Higher relative intensities of the peaks representing smaller acetylated hexobioses and diacetylated hexotrioses (H2Ac and H3Ac_2_) were observed after hydrolysis with *Cj*Man26A than with *Cj*Man5A. The shortest acetylated product resulting from the hydrolysis of SpGGM by *Cj*Man5A was acetylated hexotriose (H3Ac). This is in contrast to the acetylation profile obtained after the hydrolysis of KGM_N_ with both enzymes, where smaller acetylated mannooligosaccharides were observed after *Cj*Man5A hydrolysis. This indicates that the low native acetylation (DSac 0.09 for KGM_N_ and 0.13 for SpGGM) does not have a significant effect on substrate hydrolysis by *Cj*Man5A and *Cj*Man26A. The presence of Gal decorations and Glc units in the backbone instead appear to be the main structural factors affecting substrate recognition and enzyme action.

**Fig. 5:**
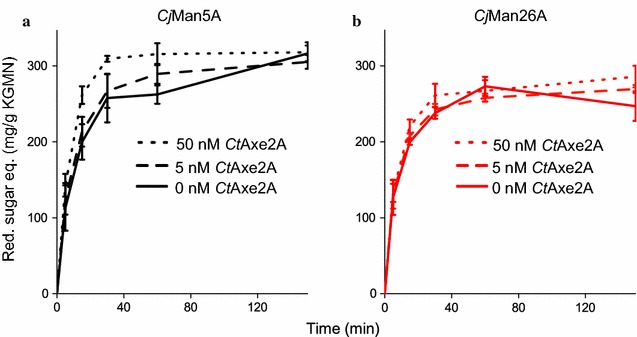
The increase in reducing sugar equivalents over 2 h in the hydrolysis of KGM_N_ with either 10 nM *Cj*Man5A (**a**) or *Cj*Man26A (**b**) with and without the addition of *Ct*Axe2A. A significant initial improvement can be observed for *Cj*Man5A at the higher acetyl esterase concentration of 50 nM. Error bars show the standard errors of the mean of triplicate measurements

To investigate whether low degrees of native acetylation which do not reduce the solubility, inhibit the action of mannanases, and whether deacetylation would improve enzymatic hydrolysis, KGM_N_ and SpGGM were incubated with either *Cj*Man5A or *Cj*Man26A, together with an acetyl esterase from *C. thermocellum*, *Ct*Axe2A. Mannanase (10 nM) supplemented with either 5, 50 nM or no *Ct*Axe2A was used in the hydrolysis reactions, and the amount of reducing sugars was monitored over time (Fig. 5). KGM_N_ hydrolysis showed a 30% increase in reducing sugar equivalents during the first hour of reaction when using 50 nM *Ct*Axe2A with *Cj*Man5A. When the lower concentration of *Ct*Axe2A (5 nM) was used, a significant increase (10%) in the amount of reducing sugars released was observed over the first hour of the reaction, indicating that the removal of acetyl groups improved the initial action of *Cj*Man5A. However, after 2 h, the reaction without the addition of *Ct*Axe2A showed a similar response to the *Ct*Axe2A-supplemented reactions. No clear improvement was observed when *Cj*Man26A was supplemented with *Ct*Axe2A, although the high addition of *Ct*Axe2A (50 nM) led to a slightly higher response, of approximately 15%, between 15 and 30 min incubation. In conclusion, supplementation with *Ct*Axe2A appeared to improve the initial hydrolysis of KGM_N_ by *Cj*Man5A.

Supplementation with *Ct*Axe2A did not significantly improve the yield of reducing sugar equivalents from SpGGM with either of the mannanases. This could be explained by the presence of galactose substituents (present in KGM_N_) which present an additional steric obstacle to efficient hydrolysis by the mannanases, even after the removal of acetyl groups.

## Discussion

Spruce is a major source of biomass in the Nordic hemisphere, and has considerable value, apart from that in pulping. Acetylated galactoglucomannan is the main hemicellulose in spruce biomass, making it interesting to evaluate its enzymatic hydrolyzability. Native konjac glucomannan harbors a glucomannan backbone, while native locust bean gum galactomannan contains galactose side groups. These three substrates represent mannans with different backbone compositions and decorations, and were therefore interesting as model substrates in the present study on the action and substrate degradation patterns of the mannanases, *Cj*Man5A and *Cj*Man26A. The oligosaccharides produced by enzymatic hydrolysis of KGM_N_ and LBG_N_ differed in terms of type, length, and amount, depending on which enzyme was applied, showing that these two mannanases have different modes of action. Generally, *Cj*Man5A produced mannotriose as the main end product, while *Cj*Man26A produced high amounts of mannose and mannobiose units. The actions of *Cj*Man5A and *Cj*Man26A on a range of mannooligosaccharides as well as *Cj*Man5A activity on KGM and LBG have been described previously by Hogg et al. [29, 30]. They found that *Cj*Man26A was 100,000 times more efficient than *Cj*Man5A in the hydrolysis of M3 oligosaccharides into M1 and M2, which is in agreement with the present results. The comparison of the cleavage patterns of *Cj*Man5A and *Cj*Man26 on KGM_N_ and LBG_N_ in the present study supports their suggestion that a secreted *Cj*Man5A plays the biological role of breaking down larger polymers into oligosaccharides, which are further hydrolyzed into mannose and mannobiose units by the membrane-bound *Cj*Man26A.

Detailed analysis with HPAEC-PAD and ESI–MS allowed us to map the product pattern on the various substrates. When comparing the hydrolysis of the homogeneous mannose backbone of LBG_N_ to the glucomannan backbone of KGM_N_, our results showed that *Cj*Man26A produced a higher quantity of the shorter M2 from LBG_N_. This was observed with ESI–MS after 24-h reactions and in HPAEC-PAD analysis at shorter incubation times. One explanation of this could be the suggested lower affinity of the − 2 subsite of *Cj*Man26A for glucose than mannose [29], making the reaction slower and incomplete on the KGM_N_ substrate. *Cj*Man5A has a more promiscuous specificity and can accommodate mannose and glucose at any of its subsites. Furthermore, hydrolysis of KGM_N_ and LBG_N_ with *Cj*Man5A showed similar product profiles for mannooligosaccharides, supporting this theory.

The present work is the first to investigate the hydrolyses of SpGGM by *Cj*Man5A and *Cj*Man26A. The results support the previously proposed differences between the two enzymes, and provide new information on the modes of action of these two enzymes. *Cj*Man26A produced more M1 and M2 from SpGGM than *Cj*Man5A, as in the case of the model substrates KGM_N_ and LBG_N_. Surprisingly, high amounts of M3 were produced by *Cj*Man26A, which had still not been further hydrolyzed after 24 h of incubation. This could possibly be explained by an overlapping peak of an unassigned oligosaccharide, confounding the HPAEC-PAD results. Another explanation of the high amount of M3 could be that acetyl groups are positioned in such a manner that they prevent further enzymatic hydrolysis. The available crystal structure of *Cj*Man26A, solved with an oligosaccharide ligand [42], allows for hypotheses regarding the effect of acetylation in certain positions. Acetylation in either position of C2 and C3 of a mannose unit in subsite − 1 appears unfavorable due to steric clashes, whereas only acetylation of C2 appears to negatively affect substrate binding in the − 2 subsite. Acetylation of the mannose residue occupying the − 1 subsite is thus a likely explanation for M3 not being further hydrolyzed into smaller products by *Cj*Man26A. The difference in SpGGM cleavage pattern, compared to KGM_N_ and LBG_N_, demonstrates the need to evaluate mannanases on a range of complex substrates to gain knowledge on the differences in mode of action and specificity.

A few studies have been carried out on the action of mannanases on acetylated substrates [43, 44]. However, comparative studies of how acetylation influences the action of different mannanases are to date lacking. One of the aims of the present study was to evaluate the influence of acetylation on hydrolysis by *Cj*Man5A and *Cj*Man26A, using both chemically and natively acetylated mannan substrates. Native acetylation is present in KGM_N_ and SpGGM on the C2 and C3 carbons of the hexose, while chemical acetylation also allows acetyl groups at the C6 position. Chemical acetylation of the KGM_N_ and LBG_N_ substrates decreased the enzymatic hydrolyzability significantly, especially in the case of the galactose-substituted LBG. Furthermore, ESI–MS spectra of enzymatically hydrolyzed KGM_A_ and LBG_A_ showed nil, or only a minor, increase in acetylated oligosaccharides as final hydrolysis products, compared to the native substrates. The lack of acetylated end products indicates that the enzymes are less able to act on the highly acetylated parts of the substrate. Bi et al. [20] also reported lower biodegradability of similarly acetylated KGM and LBG substrates, in agreement with our results. We hypothesized that the reduced hydrolyzability could be the result of *Cj*Man5A and *Cj*Man26A being unable to act on the insoluble and heavily acetylated parts of the substrates. Another possibility is that a large number of acetyl groups on the natively non-acetylated C6 position of the hexoses result in a decreased enzymatic substrate hydrolyzability. An interesting finding was that the chemical acetylation was not randomly distributed along the KGM_A_ and LBG_A_ polysaccharides. The soluble fractions exhibited nil or only a low acetyl content, similar to the acetyl content of the native substrates, implying that essentially all of the artificially added acetyl groups are added to discrete segments of the polysaccharides, rendering them insoluble.

Autohydrolysis and possible migration of the acetyl substitutions during the enzymatic hydrolysis of the natural and chemically acetylated substrates may have an impact on the results presented here. In an earlier study, acetyl migration has been observed for GGM material after 12-h incubation at 90 °C [13], but neither has the quantification nor the determination of the time course of the migration been performed. The enzymatic reactions in our study were performed at pH 7, in accordance with the enzymes’ pH dependence. Acetyl migration on galactopyranoside monomers at pH 7.6 has been observed [45], but is, however, not directly comparable with our polymeric mannan substrates due to the different stereochemistry of the C2 hydroxyl groups in mannosyl and galactosyl units (axial vs. equatorial). Moreover, during the first hour of our enzymatic reactions, the acetyl autohydrolysis and migration should be low, less than 10% according to Roslund et al. [45]. During this initial stage, a significantly lower hydrolysis rate was observed using the chemically acetylated substrates compared to the native and non-acetylated ones, and therefore it can be assumed that even if acetyl migration takes place, it has a negligible impact on our data.

Comparison of the ESI–MS spectra from enzymatically treated, natively acetylated SpGGM suggested that *Cj*Man26A was more tolerant to acetyl substituents than *Cj*Man5A, though the ESI–MS profiles of the enzymatically treated KGM_N_ substrate indicated the opposite. An improvement in the overall hydrolysis in reactions supplemented with the *Ct*Axe2A esterase was not substantial for either of the enzymes, implying that a low degree of native acetylation does not affect the end product profiles, although a minor influence on the early hydrolysis rates was observed. Structural factors, such as the presence of Gal substitutions and Glc units in the backbone, appear to be more important for substrate recognition and enzyme action.

It is important to study the actions of numerous mannanases on a variety of substrates in order to elucidate their differences in activity and product profiles. Discovering complementary activities and substrate specificities is highly interesting from an industrial perspective, not least in the context of biorefineries, where hemicellulose is an important resource. In the present study, both *Cj*Man5A and *Cj*Man26A were efficient in the deconstruction of SpGGM, producing a range of different oligosaccharides that could be evaluated regarding their potential as prebiotics or for further hydrolysis into fermentable sugars. *Cj*Man26A released smaller mono- and disaccharides and could be used for saccharification into fermentable sugars, possibly in combination with auxiliary enzymes such as β-mannosidases and α-galactosidases. *Cj*Man5A released larger oligosaccharides, and could therefore be suitable for the production of platform molecules for further use, e.g., prebiotics and macromonomers for materials applications. Native acetylation of SpGGM did not substantially inhibit the enzymatic action, and combined hydrolysis with acetyl esterases does not seem to be necessary to speed up the reaction. Chemical acetylation, on the other hand, was shown to be promising as a tool to reduce the microbial degradation of mannan-containing polysaccharides and as such is highly relevant in industrial applications where biodegradation is undesirable, e.g., in biofilms.

## Conclusions

Plant mannans can have many different configurations, with either pure mannan backbones or mixed glucomannan backbones that may also be decorated with galactose and/or acetyl groups to varying degrees. In order to shed light on the effects of these variations for two mannanases from *C. japonicus*, we enzymatically hydrolyzed a range of mannan substrates with *Cj*Man5A and *Cj*Man26A. The two mannanases exhibited strikingly different cleavage patterns on both linear and decorated mannan polysaccharides, including the naturally acetylated and industrially important SpGGM. *Cj*Man26A hydrolysis of SpGGM resulted in high amounts of mannose and mannobiose, while *Cj*Man5A produced more of the oligosaccharides, mannotriose, and mannotetraose. Hydrolysis of SpGGM with *Cj*Man26A led to the production of acetylated hexobiose, as well as diacetylated oligosaccharides, whereas the shortest acetylated oligosaccharide produced by *Cj*Man5A was hexotriose. Knowledge about the different hydrolysis-derived products will be useful for tailoring enzymatic decomposition and modification of specific hemicelluloses. Moreover, the results suggest that an increased degree of chemical acetylation strongly inhibits the action of mannanases. Synthetic acetylation could therefore be a useful strategy to reduce the degradation of mannan polymers.

## Additional files

**Additional file 1: Figure S1.** Reducing sugar equivalents over time for enzyme reactions on SpGGM.

**Additional file 2: Figure S2.** SEC profiles of KGM_N_ and LBG_N_ before and after enzymatic hydrolysis.

**Additional file 3: Figure S3.** Oligosaccharide product profiles obtained with HPAEC-PAD at three incubation times, with increasing concentrations of enzyme.

**Additional file 4: Figure S4.** Reducing sugar equivalents over time in enzymatic hydrolysis of chemically acetylated KGM_A_ and LBG_A_, compared with native KGM_N_ and LBG_N_.

**Additional file 5: Figure S5.** Oligosaccharide product profiles from HPAEC-PAD analysis of 24-h hydrolysis of the chemically acetylated substrates KGM_A_ and LBG_A_, compared with native KGM_N_ and LBG_N_.

**Additional file 6: Figure S6.** Quantification of the M1-M4 oligosaccharides produced after 24 h hydrolysis reactions from the chemically acetylated substrates KGM_A_ and LBG_A_, compared with native KGM_N_ and LBG_N_.

## Abbreviations

Ara: arabinose
DSac: degree of substitution by acetylation
ESI–MS: electrospray ionization mass spectrometry
Gal: galactose
GalA: galacturonic acid
GH: glycoside hydrolase
Glc: glucose
GlcA: glucuronic acid
HPAEC-PAD: high-performance anion exchange chromatography with pulsed amperometric detection
HPLC: high performance liquid chromatography
KGM: konjac glucomannan
LBG: locust bean gum galactomannan
M1/Man: mannose
M2: mannobiose
M3: mannotriose
M4: mannotetraose
MW: molecular weight
OLIMP: oligomeric mass profiling
Rha: rhamnose
SEC: size exclusion chromatography
SpGGM: spruce galactoglucomannan
Xyl: xylose
