# Supplementary material for: Mannanase hydrolysis of spruce galactoglucomannan focusing on the influence of acetylation on enzymatic mannan degradation
Source: Biotechnol Biofuels. 2018 Apr 19;11:114. doi: 10.1186/s13068-018-1115-y (PMC5907293; doi:10.1186/s13068-018-1115-y)
Supplement: Supplementary file 1 — Additional file 1: Figure S1. Reducing sugar equivalents over time for enzyme reactions on SpGGM. [file 13068_2018_1115_MOESM1_ESM.docx]

**
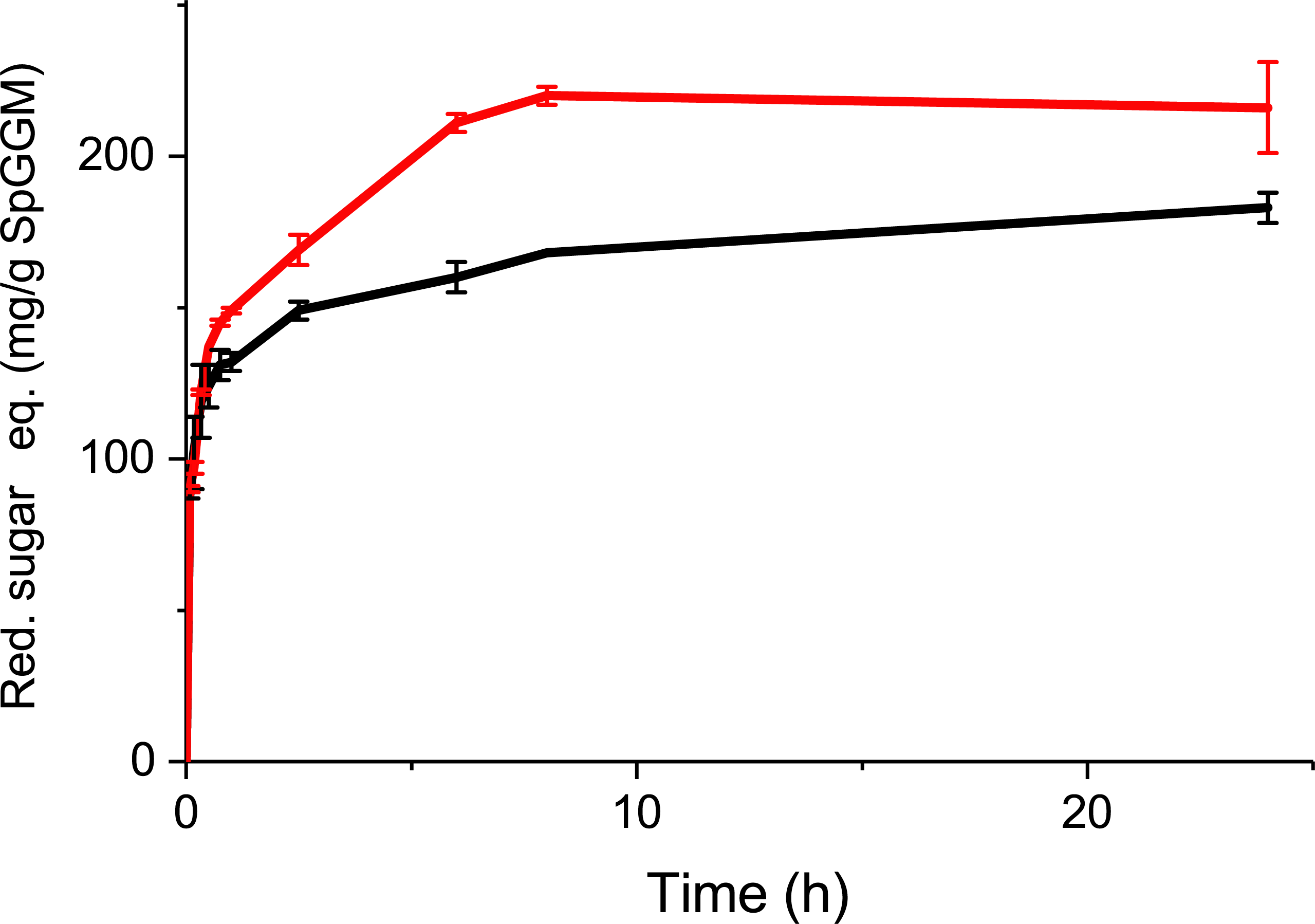
**

**Figure S1. Reducing sugar equivalents over time for CjMan5A (black) and CjMan26A (red) hydrolysis of SpGGM.** The error bars show the standard errors of the mean of triplicate measurements. The reactions contained 10 nM enzyme and 0.1 % (w/v) SpGGM.
