# Supplementary material for: Mannanase hydrolysis of spruce galactoglucomannan focusing on the influence of acetylation on enzymatic mannan degradation
Source: Biotechnol Biofuels. 2018 Apr 19;11:114. doi: 10.1186/s13068-018-1115-y (PMC5907293; doi:10.1186/s13068-018-1115-y)
Supplement: Supplementary file 2 — Additional file 2: Figure S2. SEC profiles of KGMN and LBGN before and after enzymatic hydrolysis. [file 13068_2018_1115_MOESM2_ESM.docx]

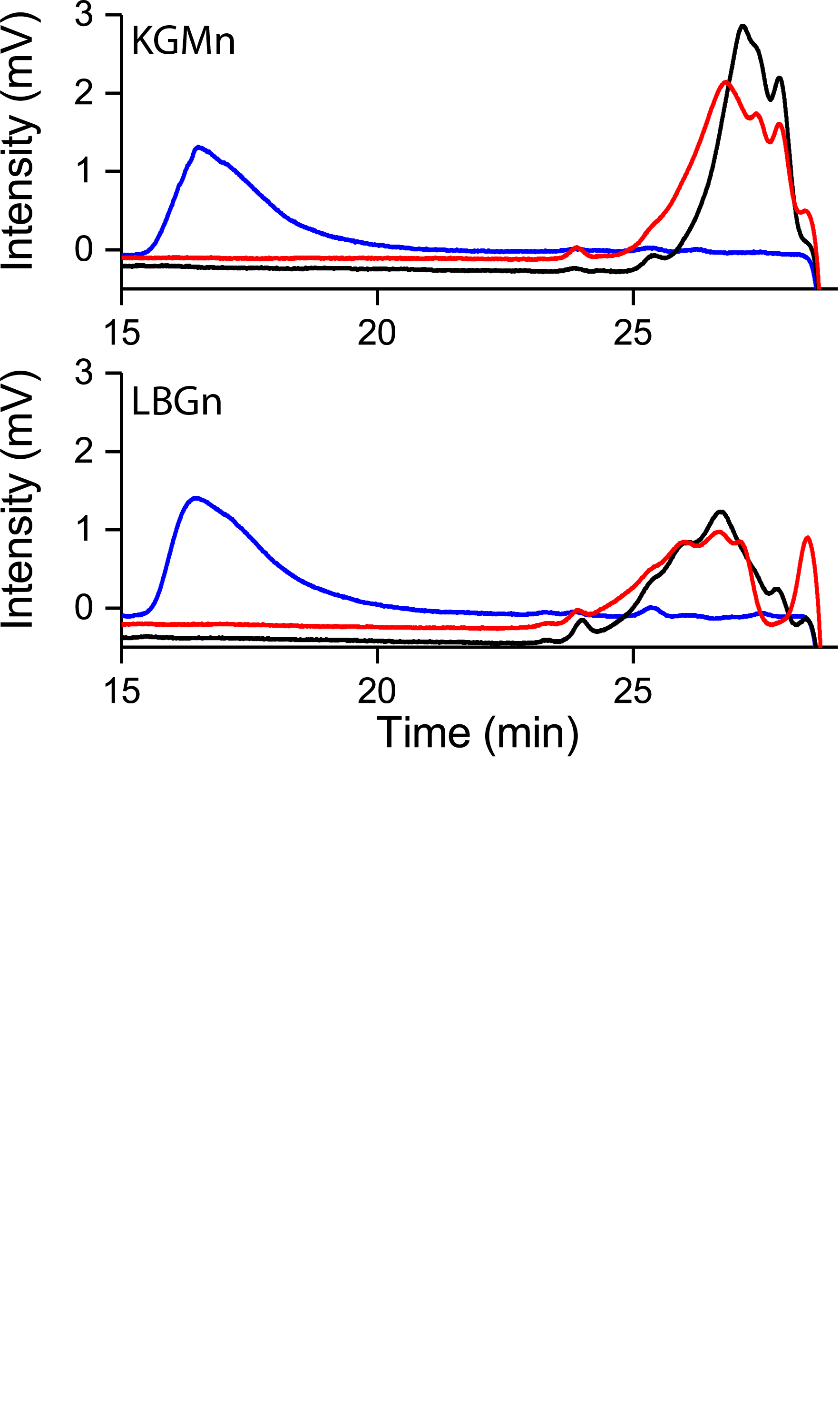


**Figure S2. KGM_N_ and LBG_N_ before (blue) and after mannanase treatment,** with either 10 nM CjMan5A (black) or CjMan26A (red), analyzed by size exclusion chromatography (SEC). SEC detects soluble substrates and products with a MW>340 Da. A disappearing substrate peak at 17 minutes (average MW~860kDa) indicates complete decomposition of the soluble fraction of the polymers into smaller oligosaccharides. CjMan5A produces oligosaccharides distributed around 900 Da from KGM_N_ and 1500 Da from LBG_N._ CjMan26A, on the other hand, produces oligosaccharides of 1000 Da from KGM_N_, and two peaks corresponding to 1800 Da and 500 Da were observed after hydrolysis of LBG_N_.
