# Supplementary material for: Mannanase hydrolysis of spruce galactoglucomannan focusing on the influence of acetylation on enzymatic mannan degradation
Source: Biotechnol Biofuels. 2018 Apr 19;11:114. doi: 10.1186/s13068-018-1115-y (PMC5907293; doi:10.1186/s13068-018-1115-y)
Supplement: Supplementary file 3 — Additional file 3: Figure S3. Oligosaccharide product profiles obtained with HPAEC-PAD at three incubation times, with increasing concentrations of enzyme. [file 13068_2018_1115_MOESM3_ESM.docx]

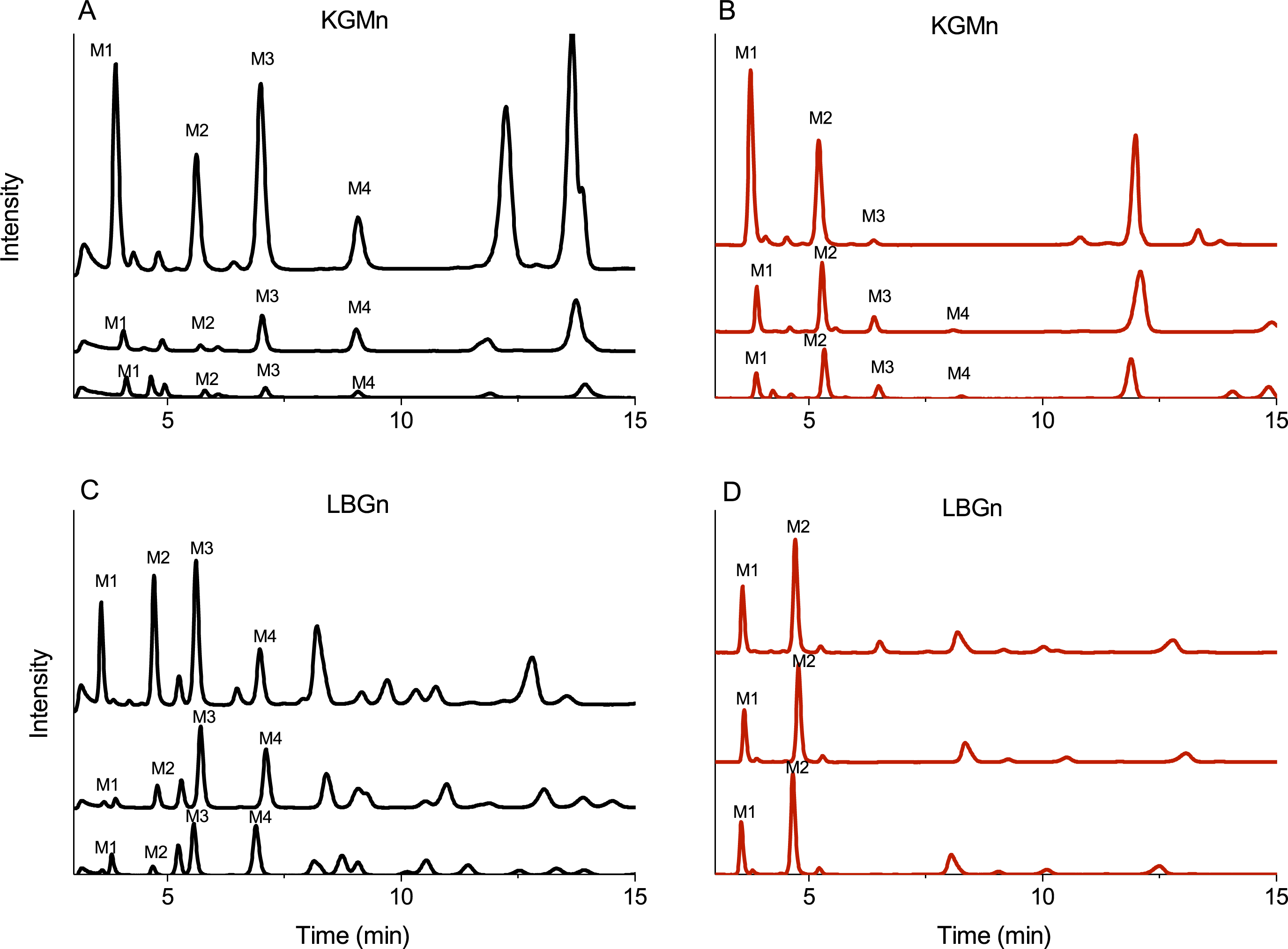


**Figure S3. Oligosaccharide product profiles obtained with HPAEC-PAD** for: A) CjMan5A and KGM_N_, B) CjMan26A and KGM_N_, C) CjMan5A and LBG_N_ and D) CjMan26A and LBG_N_, at three incubation times (t_1_, t_2_ and t_3_, from bottom to top), and increasing concentrations of enzyme. For CjMan5A reactions, t_1_=10 min, 25 nM enzyme concentration, t_2_=30 min, 25 nM enzyme concentration and t_3_=24 hours, 100 nM enzyme concentration. For reactions with CjMan26A, t_1_=5 min, 10 nM enzyme concentration, t_2_=30 min, 25 nM enzyme concentration and t_3_=24 h, 100 nM enzyme concentration. As expected, an increase in the production of shorter mannooligosaccharides is observed with time.
