# Supplementary material for: Mannanase hydrolysis of spruce galactoglucomannan focusing on the influence of acetylation on enzymatic mannan degradation
Source: Biotechnol Biofuels. 2018 Apr 19;11:114. doi: 10.1186/s13068-018-1115-y (PMC5907293; doi:10.1186/s13068-018-1115-y)
Supplement: Supplementary file 4 — Additional file 4: Figure S4. Reducing sugar equivalents over time in enzymatic hydrolysis of chemically acetylated KGMA and LBGA, compared with native KGMN and LBGN. [file 13068_2018_1115_MOESM4_ESM.docx]

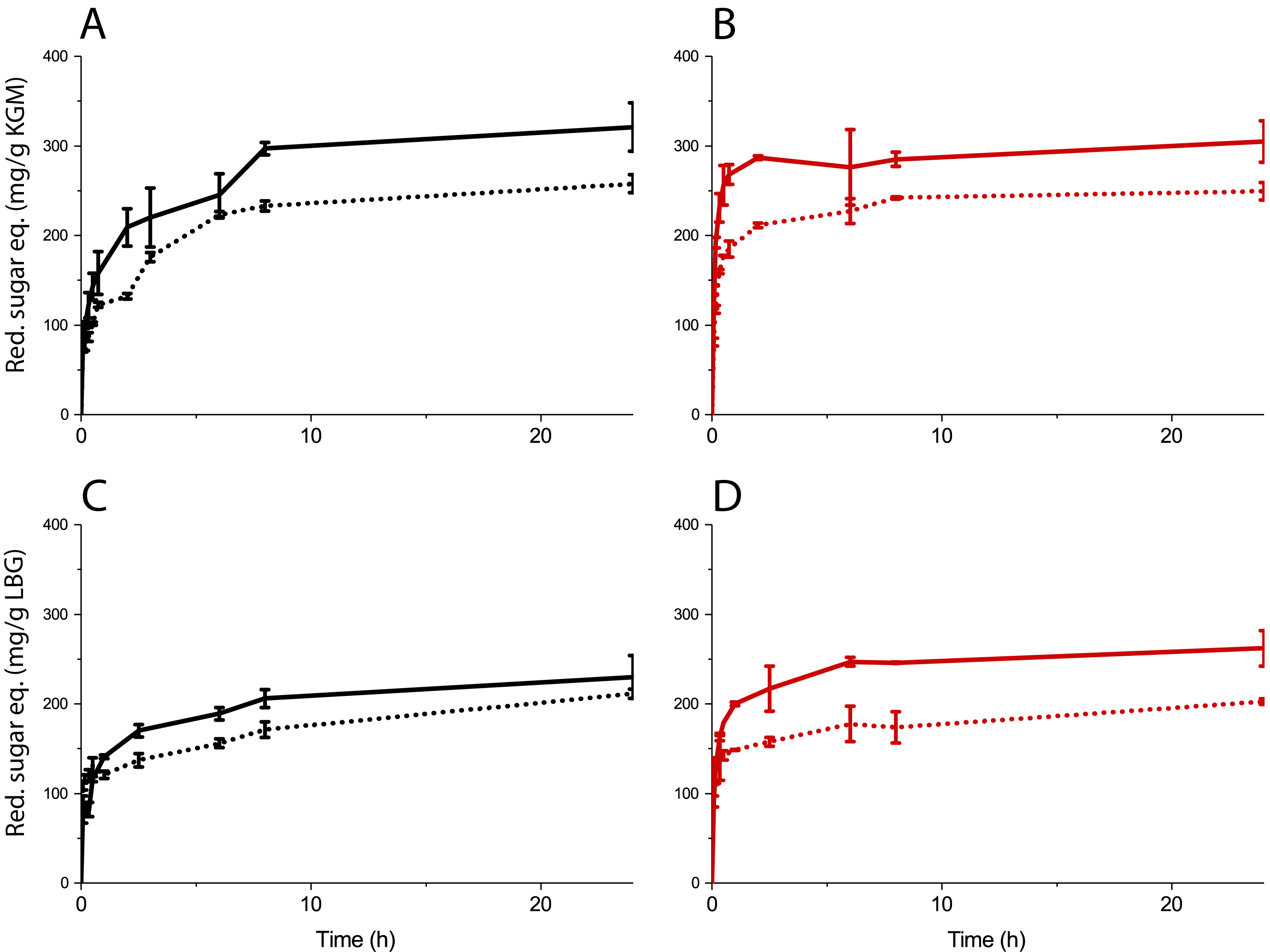


**Figure S4. Reducing sugar equivalents over time for CjMan5A (black) and CjMan26A (red) reactions** with A) & B) KGM_A_ (dashed line) versus KGM_N_ (solid line), C) & D) LBG_A_ (dashed line) versus LBG_N_ (solid line). The error bars show standard errors of the mean of triplicate measurements. The chemically acetylated substrates have a DSac 0.7-0.8. The hydrolysis reactions contained 10 nM enzyme (KGM_N_, KGM_A_, LBG_N_) or 100 nM enzyme (LBG_A_) and 0.1 % (w/v) mannan substrate. The results show a significant decrease in reducing sugar equivalents upon chemical acetylation of the mannan substrates.
