# Supplementary material for: Mannanase hydrolysis of spruce galactoglucomannan focusing on the influence of acetylation on enzymatic mannan degradation
Source: Biotechnol Biofuels. 2018 Apr 19;11:114. doi: 10.1186/s13068-018-1115-y (PMC5907293; doi:10.1186/s13068-018-1115-y)
Supplement: Supplementary file 5 — Additional file 5: Figure S5. Oligosaccharide product profiles from HPAEC-PAD analysis of 24-h hydrolysis of the chemically acetylated substrates KGMA and LBGA, compared with native KGMN and LBGN. [file 13068_2018_1115_MOESM5_ESM.docx]

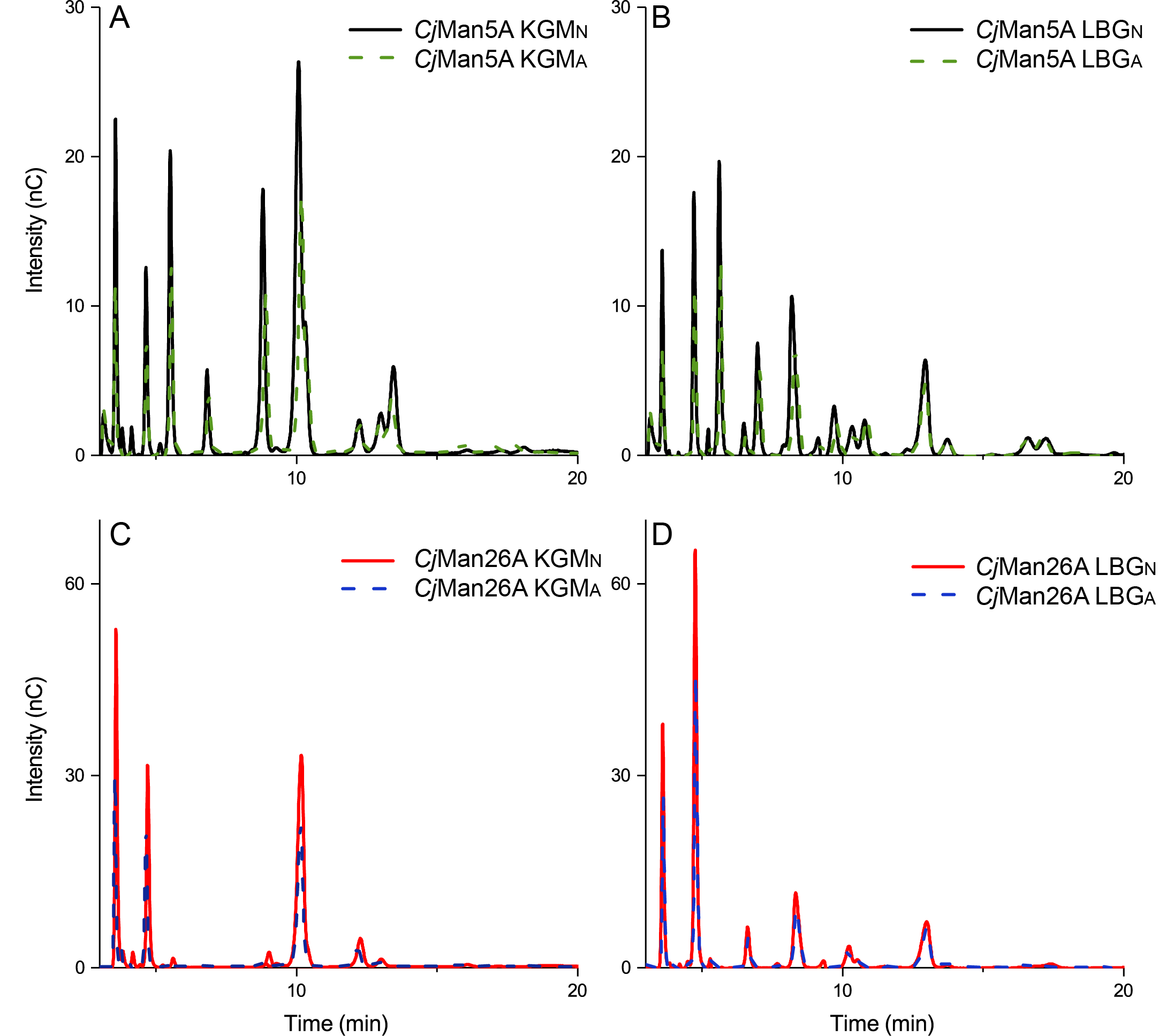


**Figure S5. Oligosaccharide product profiles obtained with HPAEC-PAD** for: A) *Cj*Man5A and KGM, B) *Cj*Man5A and LBG, C) *Cj*Man26A and KGM and D) *Cj*Man26A and LBG, after 24-h hydrolysis. The hydrolysis reactions contained 100 nM enzyme and 0.1 % (w/v) mannan substrate. The results show a substantial decrease in intensity upon chemical acetylation of the mannan substrates, although the shape of the profiles remains the same.
