# Supplementary material for: Mannanase hydrolysis of spruce galactoglucomannan focusing on the influence of acetylation on enzymatic mannan degradation
Source: Biotechnol Biofuels. 2018 Apr 19;11:114. doi: 10.1186/s13068-018-1115-y (PMC5907293; doi:10.1186/s13068-018-1115-y)
Supplement: Supplementary file 6 — Additional file 6: Figure S6. Quantification of the M1-M4 oligosaccharides produced after 24 h hydrolysis reactions from the chemically acetylated substrates KGMA and LBGA, compared with native KGMN and LBGN. [file 13068_2018_1115_MOESM6_ESM.docx]

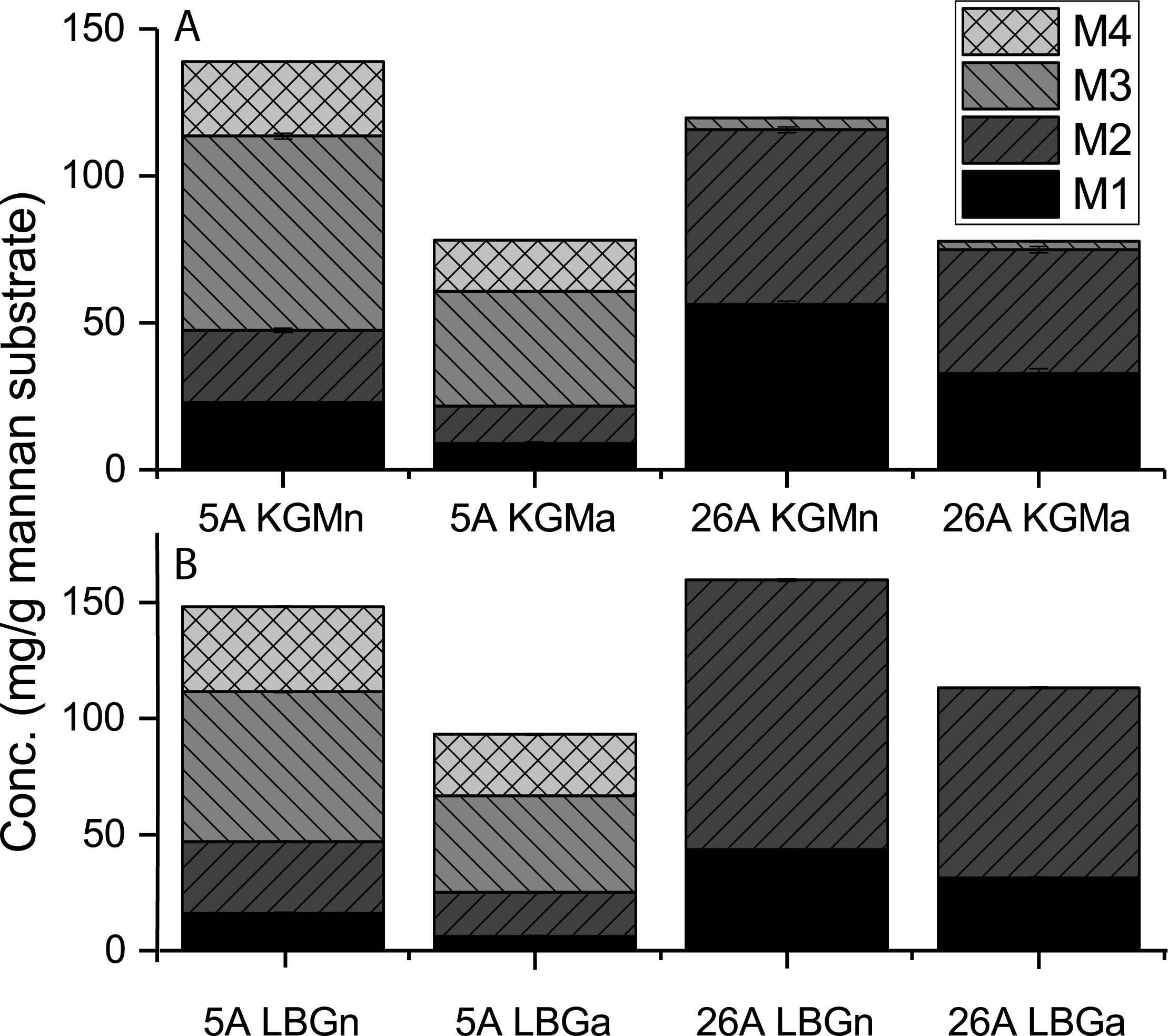


**Figure S6. HPAEC-PAD quantification of M1-M4 products** from chemically acetylated substrates (KGM_A_ and LBG_A_) and native substrates (KGM_N_ and KBG_N_) after 24 hours of enzymatic hydrolysis with *Cj*Man5A or *Cj*Man26A. The error bars show standard errors of the mean of duplicate measurements. The results show a significant decrease in final M1-M4 concentration upon chemical acetylation of the mannan substrates.
